# Ecotoxicity screening evaluation of selected pharmaceuticals and their transformation products towards various organisms

**DOI:** 10.1007/s11356-020-08881-3

**Published:** 2020-05-01

**Authors:** Łukasz Grabarczyk, Ewa Mulkiewicz, Stefan Stolte, Alan Puckowski, Magdalena Pazda, Piotr Stepnowski, Anna Białk-Bielińska

**Affiliations:** 1 Department of Environmental Analysis, Faculty of Chemistry, University of Gdańsk, ul. Wita Stwosza 63, 80-308 Gdańsk, Poland; 2 Institute of Water Chemistry, Technische Universität Dresden, 01062 Dresden, Germany

**Keywords:** Pharmaceuticals, Transformation products, Metabolites, Ecotoxicity, Toxicity, Environment, Biotests

## Abstract

The intensive development of medical science has led to an increase in the availability and use of pharmaceutical products. However, nowadays, most of scientific attention has been paid to the native forms of pharmaceuticals, while the transformation products (TPs) of these substances, understood herein as metabolites, degradation products, and selected enantiomers, remain largely unexplored in terms of their characterization, presence, fate and effects within the natural environment. Therefore, the main aim of this study was to evaluate the toxicity of seven native compounds belonging to different therapeutic groups (non-steroidal anti-inflammatory drugs, opioid analgesics, beta-blockers, antibacterial and anti-epileptic drugs), along with the toxicity of their 13 most important TPs. For this purpose, an ecotoxicological test battery, consisting of five organisms of different biological organization was used. The obtained data shows that, in general, the toxicity of TPs to the tested organisms was similar or lower compared to their parent compounds. However, for example, significantly higher toxicity of the R form of ibuprofen to algae and duckweed, as well as a higher toxicity of the R form of naproxen to luminescent bacteria, was observed, proving that the risk associated with the presence of drug TPs in the environment should not be neglected.

**Electronic supplementary material:**

The online version of this article (10.1007/s11356-020-08881-3) contains supplementary material, which is available to authorized users.

## Introduction

The intensive development of medical science has led to an increase in the availability and use of pharmaceutical products (Jjemba 2006; Bu et al. 2016). Due to this, significant amounts of substances of this group are detected in various environmental compartments. Particularly, the aquatic environment constitutes a reservoir of drugs, used in both human and veterinary medicine. Given the division of drugs by application, the sources of these substances in the environment differ. The human pharmaceuticals are mainly introduced by discharging effluent water with unmetabolized and unused drugs from hospitals, households, and pharmacological industry. These waters usually end up in wastewater treatment plants (WWTPs), where depending on the technology the drugs are more or less degraded (Verlicchi et al. 2012; Sui et al. 2015; Chen et al. 2016; Pereira et al. 2017). In turn, the veterinary pharmaceuticals are most often used as food additives, for production of both terrestrial and aquatic animals, and play different roles (e.g., prophylactic, curative, growth support) in the target animal organisms. The unmetabolized drugs and their metabolites are released with feces and urine and make their way into aquatic compartments via, i.e. leaching, surface runoff from contaminated manure (used as fertilizer), and direct contamination in aquaculture applications (Boxall et al. 2004; Sarmah et al. 2006; Kümmerer 2009; Białk-Bielińska et al. 2011; Ying et al. 2013). As a result, the emerging contaminants, such as pharmaceuticals, introduced into environment may cause negative effects on ecosystems. Available literature data on the harmfulness and occurrence of pharmaceuticals in the environment indicate the need for their environmental risk assessment (Han and Lee 2017; Desbiolles et al. 2018).

However, nowadays, most of scientific attention has been paid to the native forms of pharmaceuticals, while the transformation products (TPs) of these substances, understood herein as metabolites excreted from the organisms as well as their degradation products resulting from hydrolysis, photolysis, and biodegradation, remain largely unexplored in terms of their characterization, presence, fate, and effects that include an impact on the natural environment and human health (Mompelat et al. 2009; Fatta-Kassinos et al. 2011; Wilkinson et al. 2017; Bottoni and Caroli 2018; Brown and Wong 2018). Despite the evidence for occurrence of TPs of pharmaceuticals most often detected in the environment (Mompelat et al. 2009; Fatta-Kassinos et al. 2011; Verlicchi et al. 2012; Lonappan et al. 2016), a substantial gap in knowledge on the potential ecotoxicological effects of these substances exists. Fatta-Kassinos et al. (2011) presented a review of the topic including TPs of various antibiotics, non-steroidal anti-inflammatory drugs (NSAIDs), and beta-blockers, stressing a need for their ecotoxicological assessment, since after formation TPs can, in some cases, be not only more toxic but also more stable and abundant in the receiving environments. Nevertheless, some examples of TPs, which show similar or higher biological activity relative to their native forms, can be found in literature. In the case of antibacterial drugs, studies on sulfonamides confirm the abovementioned concerns, reporting lower biological activity of TPs in most cases, however with some exceptions, e.g. TP of sulfanilamide was found to elicit similar toxicity towards algae and aquatic plants as its parent compounds (Isidori et al. 2005a; Kim et al. 2007; García-Galán et al. 2008; Białk-Bielińska et al. 2017). Studies on NSAIDs, such as diclofenac (DIC), show a higher toxicity of its TPs towards algae (Lonappan et al. 2016), but no acute toxicity towards marine bacteria and crustaceans (Osorio et al. 2016; Ma et al. 2017). Nevertheless, TPs of both groups of substances were detected in the environment and their mixture toxicity is determined as additive and possibly synergistic (Lonappan et al. 2016; Osorio et al. 2016; Białk-Bielińska et al. 2017). Also, in the case of another NSAID, naproxen (NAP), four of its TPs were found more toxic towards algae, rotifers, and micro crustaceans in both acute and chronic tests (Isidori et al. 2005b). Furthermore, to give some examples of other drug groups: *O*-desmethyltramadol (*O*-DES-TRA), the metabolite of an opioid analgesic tramadol (TRA), was found to be a stronger inhibitor of opioid receptors, and metoprolol acid (MET-ACID), a beta-blocker TP, was also found slightly more toxic than the parent compound towards three species of fungi (Jaén-Gil et al. 2019). The occurrence of pharmaceutical TPs in the environment and their potential biological activity indicate the need to extend the environmental risk assessment with ecotoxicological tests for these substances (Han and Lee 2017).

Therefore, the main aim of our study was to evaluate the toxicity of selected TPs of different pharmaceuticals, which are commonly detected in many aquatic environments at relatively high concentrations in comparison to other therapeutic compounds. The list of selected TPs is presented in Table 1, the choice of which was based on available data on their excretion rate, biological activity, stability, and/or available literature data proving their occurrence in the environment.

**Table 1:**
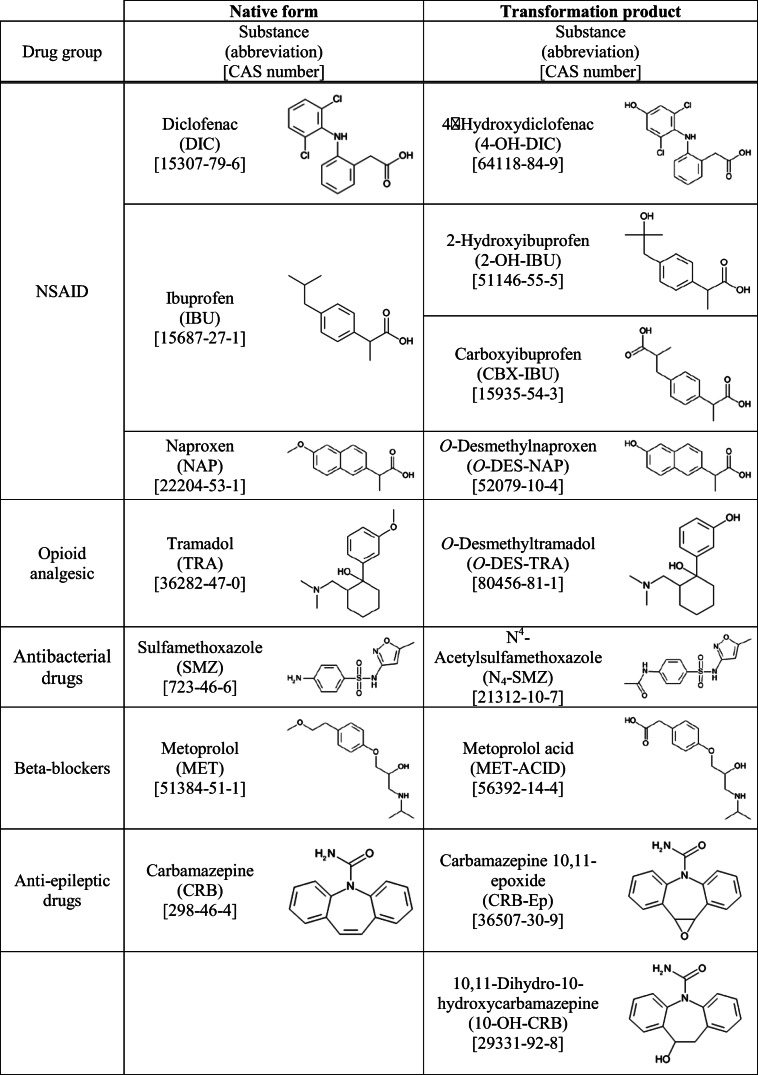
Investigated native forms (NF) of pharmaceuticals and their TPs

As presented above, the selected pharmaceuticals belong to different therapeutic groups: NSAIDs, opioid analgesics, beta-blockers, and antibacterial and anti-epileptic drugs. All of the native compounds are well-established contaminants of concern for the environment (Desbiolles et al. 2018). NSAIDs constitute anti-inflammatory, antipyretic, and analgesic agents, commonly used and easily accessible worldwide. They are considered safe due to the rare occurrence of side effects, mainly in children and the elderly (Sandilands and Bateman 2016; Terzi et al. 2017). Diclofenac, naproxen, and ibuprofen (IBU) were selected from this drug group along with their four TPs (Table 1), due to confirmed presence in WWTP effluents at concentrations from ng L^−1^ to μg L^−1^ levels (Lonappan et al. 2016; Han and Lee 2017; Wilkinson et al. 2017), as well as due to the abovementioned ecotoxicological data (Isidori et al. 2005b; Lonappan et al. 2016). A substance of similar function (opioid analgesic), TRA, was selected because of its wide use (and apparent abuse), along with its primary metabolite *O*-DES-TRA. The compounds are frequently detected at around 100 ng L^−1^ levels (Chen et al. 2016; Han and Lee 2017). Despite seemingly low concentrations, the high biological activity of both the parent compound and metabolite (Hafezi Moghadam et al. 2016; Lagard et al. 2016) justify this choice. Furthermore, *O*-DES-TRA is prevalent in water and shows tendency for bioaccumulation (Archer et al. 2017). From the group of antibacterial drugs, sulfamethoxazole (SMZ) and its main human metabolite N^4^-acetylsulfamethoxazole (N_4_-SMZ) were selected. Sulfonamides represent a highly versatile and cheap group of antibacterial agents, useful in treatment for both gram-positive and gram-negative bacteria in human and veterinary medicine (Białk-Bielińska et al. 2011). The presence of N_4_-SMZ in the environment is confirmed (at levels from few hundred of ng L^−1^ up to around 2 μg L^−1^). The TP is identified in WWTP effluents, and the potential environmental risk caused by this metabolite is higher than that of the parent compound (Han and Lee 2017; Vila-Costa et al. 2017; Brown and Wong 2018). Interestingly, N^4^-acetylated sulfamethoxazole can be transformed into the parent compound during the storage of manure and in wastewater treatment (Göbel et al. 2004; García-Galán et al. 2008). Another pair of substances selected for research is metoprolol (MET) and its main metabolite/biodegradation product MET-ACID. Beta-blockers are recommended for the treatment of hypertension in people with heart disease, and MET represents one of the first beta-blockers, which found wide application in medicine (Ahad et al. 2015; Vale 2016). The selected metabolite (MET-ACID) is frequently found alongside MET in environmental compartments, but at 10 times higher concentrations (up to 2.5 μg L^−1^) (Petrie et al. 2014; Evgenidou et al. 2015). This is related to the fact that MET-ACID is also a TP of other beta-blockers (e.g. atenolol) and is more recalcitrant to biodegradation than the parent compound (Evgenidou et al. 2015). Furthermore, as mentioned before, MET-ACID shows similar ecotoxicological effects as MET (Jaén-Gil et al. 2019). The last group of selected pharmaceuticals is anti-epileptic drugs, namely carbamazepine (CRB), together with its two TPs: carbamazepine 10,11-epoxide (CRB-Ep) and 10,11-dihydro-10-hydroxycarbamazepine (10-OH-CRB). Due to the high stability of CRB, this substance is one of the most frequently determined pharmaceuticals in the environment, at concentrations up to 2 mg L^−1^ (Camacho-Muñoz et al. 2010; Rajendran and Sen 2018). It was also found to be toxic towards various aquatic non-target organisms, such as bacteria, algae, and fish (Kim et al. 2007; Camacho-Muñoz et al. 2010; Rajendran and Sen 2018). Both selected TPs of CRB show similar or higher toxicity towards marine bacteria than the parent compound (Brezina et al. 2017). Furthermore, small losses (up to 20%) were observed in water treatment process accounting for their stability (Brezina et al. 2017). The concentrations of CRB-Ep and 10-OH-CRB in the environment were found to be up to 4 μg L^−1^ (Brezina et al. 2017; Han and Lee 2017; Bottoni and Caroli 2018; Sharma et al. 2018). Additionally, both TPs show a tendency for bioaccumulation (Bottoni and Caroli 2018).

Summing up, a hypothesis is stated that there is a potential high bioactivity (consequently toxicity and ecotoxicity) of several groups of pharmaceuticals and TPs of thereof, which are being released and further transformed in the environment and which could pose an even greater risk than the parent compounds. Therefore, seven native forms of pharmaceuticals and nine of their transformation products were subjected to a flexible ecotoxicological test battery, consisting of five organisms of different biological organization. Among the aquatic organisms, luminescent bacteria (*Vibrio fischeri*), green algae (*Raphidocelis subcapitata*), duckweed (*Lemna minor*), and daphnia (*Daphnia magna*) were selected. Additionally, as a representative of terrestrial organisms, a soil bacteria (*Arthrobacter globiformis*) was chosen.

An additional part of the current study was the preliminary evaluation of the toxicity of the enantiomers of ibuprofen and naproxen as it is commonly known that some of the drugs available on the market are in the form of a racemic mixture (equimolar mix of two enantiomers), while the others contain only single enantiomers. Moreover, it must be highlighted that chiral inversion, which may occur during metabolism in the body, or biological reactions occurring in the process of wastewater treatment, may result in enrichment in one of the enantiomers. This also proves that different enantiomeric forms might be also considered as specific TPs of selected pharmaceuticals. Documented differences in enantiomer toxicity within the human body (Kasprzyk-Hordern 2010) may also suggest different toxicity to organisms in the environment. Available literature data indicate significantly different toxic effects of chiral forms of the same drug observed in various species of aquatic organisms (Stanley et al. 2006; Stanley et al. 2007; De Andrés et al. 2009; Ribeiro et al. 2011; Sanganyado et al. 2017). Therefore, ecotoxicological data obtained based on tests using racemic mixtures may be the reason for underestimating the real risk associated with the introduction of enantiomers of drugs not necessarily in the same amounts to the environment. However, since such research is very limited, it was emphasized that more of such data was needed to establish these differences (Nilos et al. 2011; Ribeiro et al. 2011; Kasprzyk-Hordern 2010; Sanganyado et al. 2017). Therefore, both forms of compounds: R-naproxen - CAS: 23979-41-1 (R-NAP), S-naproxen - CAS: 22204-53-1 (S-NAP), R-ibuprofen - CAS: 51146-57-7 (R-IBU), and S-ibuprofen - CAS: 51146-56-6 (S-IBU) were studied under the same conditions as other selected TPs.

## Materials and methods

### Chemicals

CRB, TRA, NAP, DIC, IBU, SMZ, MET, *O*-DES-NAP, *O*-DES-TRA, 4-OH-DIC, 2-OH-IBU, CBX-IBU, N_4_-SMZ, and salts used for the preparation of the test media were purchased from Sigma–Aldrich (Steinheim, Germany). CRB-Ep, 10-OH-CRB, MET-ACID, and R-NAP were obtained from Toronto Research Chemicals (North York, Canada). S-NAP was sourced from Cayman Chemical Company whereas R- and S-IBU were from Santa Cruz Biotechnology.

All solutions were prepared immediately before the test in a suitable test medium. To improve the solubility of studied compounds (with an exception of DIC, TRA, MET, and *O*-DES-TRA) in the test media, an addition of organic solvent (acetone or DMSO) was necessary. In order to confirm that the addition of an organic solvent does not cause negative effects, solvent controls were also included in each test. However, no toxic effect was observed for the highest concentration of organic solvent used.

### Ecotoxicological tests

All tests were carried out in accordance with the OECD or ISO guidelines. In general the experimental part was divided into three main steps: (i) determination of the toxicity of the native forms of pharmaceuticals (their racemic mixtures), (ii) toxicity evaluation of the selected TPs of these pharmaceuticals, and (iii) preliminary toxicity assessment of the enantiomers of selected pharmaceuticals. For each assay, range-finding tests were conducted, in order to determine the range of concentrations for the definitive tests. The main tests were repeated three times in at least two parallel replicates, with a minimum of five concentrations for native forms of the selected pharmaceuticals (including their enantiomers). The exact number of replicates and concentrations tested was included in the description of each test. The highest tested analyte concentration was 100 mg L^−1^, due to the fact that according to the EC-Directive 93/67/EEC (European Commission 1993), substances with EC_50_ values higher than 100 mg L^−1^ are not considered as harmful to aquatic organisms. However, TPs were tested in a different manner; first, the tests were carried out at the limit concentration of 100 mg L^−1^. When the observed inhibition was higher than 50%, subsequent tests (due to their high costs) were performed at one concentration point-equivalent to the EC_50_ of the native form. Such approach was applied in order to observe the relative toxicity of the selected TP and its native form. Finally, the toxicity of the selected enantiomers of IBU and NAP was assessed towards the following organisms: *V. fischeri*, *D. magna*, *R. subcapitata*, and *L. minor*.

The reliability of each test was confirmed by testing the appropriate reference substances (3,5-dichlorophenol in case of algae and duckweed; potassium dichromate in case of luminescent bacteria and daphnia). Each of the tests performed met the validation criteria described in the adequate guidelines. Dose-response curves were fitted using a linear logistic or logistic model (the model giving the best fit was chosen) (https://cran.r-project.org/web/packages/drfit/drfit.pdf). The mathematic formulas applied for this purpose are presented and described in the Supplementary Material. The EC_50_ values were given since log EC_50_ is a model parameter in the logistic as well as in linear logistic model. Calculations were carried out using drfit package with R language and environment for statistical computing (http://www.r-project.org) (R Core Team 2014).

#### *Vibrio fischeri*

The *V. fischeri* luminescence inhibition assay was performed in accordance with ISO11348-3:2007 guideline (ISO 11348-3:2007(en) 2007) using commercially available LCK 482 test kit (Dr. Lange GmbH, Germany). Within each test, at least four controls (2% NaCl in phosphate buffer), four positive controls (7.5% NaCl in phosphate buffer), and eight dilutions of the tested substance were used in two parallel replicates. Stock solutions and dilutions were prepared in 2% NaCl in 0.02 M phosphate buffer, pH 7. The freeze-dried bacteria were rehydrated prior to testing in a reactivation solution. Culture suspensions and diluted samples were pre-incubated at 15 °C for 15 min. After the initial luminescence was measured, 0.5 mL of the culture suspension was mixed with the same volume of a diluted sample. The final bioluminescence was measured after 30 min. Incubation was kept at 15 °C (LUMIStox 300 meter, Dr. Lange GmbH, Berlin, Germany). The relative toxicity of the samples was expressed as a percentage of luminescence inhibition compared to the controls.

#### *Daphnia magna*

The *D. magna* acute immobilization test was performed using the commercially available DAPHTOXKIT F (MicroBioTest Incorporation, Gent, Belgium), which is developed in accordance with the OECD 202 guideline (OECD 202 2004). Stock solutions and dilutions were prepared in the test medium, which consisted of NaHCO_3_ (67.75 mg L^−1^), CaCl_2_ × 2H_2_O (294 mg L^−1^), MgSO_4_ × 7H_2_O (123.25 mg L^−1^), and KCl (5.75 mg L^−1^). The pH was checked at the beginning and at the end of the test and was within the range from 6 to 8. Three days before the test, ephippia were transferred into petri dishes with the test medium and incubated for 72 h at 22 °C (± 1 °C) under irradiation of 114 μmol photons m^−2^ s^−1^ (6000 lx) for hatching. Five pre-fed test organisms were incubated with the studied compounds diluted in 10 mL of test media in glass test vessels at 20 °C (± 1 °C) in darkness for 48 h. Each test consisted of a control and five different concentrations of the studied compound in four parallel replicates. In order to achieve a full dose-response relationship, the concentration series covered the range of 0–100% immobilization. The number of immobilized or dead organisms was checked after 24 and 48 h. The negative effect of exposure to the tested compounds was expressed by the number of immobilized organisms compared to the controls.

#### *Raphidocelis subcapitata*

The *R. subcapitata* reproduction inhibition test was carried out in accordance with the OECD 201 guideline (OECD 201 2011). Test organisms were provided from Algal Culture Collection (Universität Göttingen, Germany). The stock culture was grown in OECD 201 medium (Table 1S in the Supplementary Material) in a light-dark cycle (16 h to 8 h) at 23 °C during the light period and at 20 °C during the dark period. The light intensity was 142.5 μmol photons m^−2^ s^−1^ (7500 lx). The light-dark cycle was different from the OECD guidelines, and it was introduced to imitate natural growth conditions. In order to ensure that the algae are in the exponential growth phase when used to inoculate the test solutions, the stock culture was diluted three times a week. The test was carried out in 25 mL suspension cell culture flasks (Nunc) (working volume 10 mL). All operations were performed under sterile conditions. The initial cell concentration was 5 × 10^4^ cells mL^−1^. Eight different concentrations of every compound in three replicates and a minimum of six controls were used in each test. The concentration series covered the range of 5–85% inhibition of algal growth rate. The pH was checked at the beginning and at the end of the test and was in the range from 6 to 8. The test flasks were incubated on a shaker with a speed of approx. 75 rpm at the temperature and light conditions described above. Growth inhibition was calculated using the cell counts of the treated samples in relation to the untreated controls after 72 h of exposure. The cells were counted with the use of CASY TT Cell counter & analyzer.

#### *Lemna minor*

The *L. minor* growth inhibition test was carried out in accordance with the OECD 221 guidelines (OECD 221 2006). Duckweed was grown in Erlenmeyer flasks in 150 mL of Steinberg medium (Table 2S in the Supplementary Material) in a climate chamber at 25 °C (± 1 °C) under irradiation of 114 μmol photons m^−2^ s^−1^ (6000 lx) and a humidity of 60%. One week before the assay, the inoculum cultures were prepared; eight healthy plants were placed into a new portion of the medium. Two days before the test, the plants were transferred to a fresh medium, in order to supplement nutrients and to eliminate plant metabolites. The experiments were performed in six-well plates under the conditions described above. The pH value of the Steinberg medium and all solutions was 5.5 (± 0.5). Every test included six different concentrations of compound and six controls in three replicates. The test started with one plant consisting of three fronds and the measured endpoint was the inhibition of growth rate determined by comparing the frond area (mm^2^) for the treated plants and untreated controls. The frond area was measured using a set consisting of a Photocamera IDS UJ-1460LE-C-HQ (iDS, Germany) and software WinDias 3 (Delta-T Devices Ltd., Germany).

#### *Arthrobacter globiformis*

The test strain was obtained from the German Collection of Microorganisms (DSMZ). A version of the test without soil was performed. Bacteria was grown in a sterile medium containing 10 g L^−1^ peptone from casein, 5 g L^−1^ yeast extract, 5 g L^−1^ glucose, and 5 g L^−1^ NaCl. For the test, the medium was diluted in a ratio of 1:3 in water. The inoculum was the bacteria in a log-growth phase. Cell density was adjusted to 1 × 10^8^ cells mL^−1^, which corresponds to an optical density (OD_600nm_) of 0.4. The test was performed in 24-well microplates. Each well contained 1 mL of solution of the tested compound in water and 1 mL of bacterial inoculum. Next, the plates were incubated for 2 h at 30 °C on a horizontal shaker with a speed of 100 rpm. To each well, a portion of 0.6 mL of the redox active dye resazurine (45 mg L^−1^) dissolved in buffer was added. The microplates were further incubated for 1 h (30 °C, 100 rpm). To stop the reduction process, the plates were centrifuged at 3600*g* for 10 min. Aliquots (300 μl) of supernatant from each well were immediately transferred to the wells of a 96-well microplate in triplicates. Finally, the dehydrogenase activity was determined by measuring the formation of resorufin from resazurine using a fluorimeter (em. 535 nm, exc. 590 nm). Dehydrogenase inhibition was calculated using Eq. 1.

1 $$ I=100-\left[\frac{F_{BTS}-{F}_{TS}}{F_{BNC}-{F}_{NC}}\right]\times 100\ \left( in\%\right) $$

where F_BTS_ is the measured fluorescence of the blank (medium) with the tested substance, F_TS_ is the measured fluorescence of the sample, F_BNC_ is the measured fluorescence of the blank, and F_NC_ is the measured fluorescence of the negative control (with bacteria).

## Results and discussion

The ecotoxicological risk of the selected drugs and their most frequently occurring TPs was evaluated by determining their toxicity to selected aquatic and terrestrial organisms. As a result of the conducted research, new ecotoxicological data for not only the selected pharmaceuticals, but also their transformation products, has been provided. Such results may pose a significant role in the determination of the environmental risk of these substances in the future. The obtained toxicity data for the native forms of pharmaceuticals are presented in Table 2. All obtained dose-response curves as well as specific parameters describing dose-response curves are presented in Figs. 1S–8S and in Tables 3S–10S in the Supplementary Material.

**Table 2:** EC_50_ values (mg L^−1^) (with confidence interval 2.5–97.5%) determined for native forms of pharmaceuticals in ecotoxicity tests selected for the study

Compound	*V. fischeri*	*D. magna*	*L. minor*	*R. subcapitata*	*A. globiformis*
DIC	11.62 (11.26 – 11.99)	59.09 (55.23 – 63.19)	16.52 (15.28 – 17.61)	NA	> 100
IBU	14.97 (14.51 – 15.43)	50.07 (47.52 – 52.82)	13.25 (12.14 – 14.48)	93.26 (77.78 – 122.07)	> 100
NAP	25.17 (23.65 – 26.70)	74.39 (70.06 – 78.87)	20.70 (18.87 – 23.73)	27.10 (24.82 – 29.71)	> 100
SMZ	51.77 (49.61 – 53.95)	42.74 (40.20 – 45.48)	3.07 (2.63 – 3.61)	4.36 (3.46 – 5.52)	> 100
CRB	> 100	> 100	50.17 (46.45 – 53.81)	> 100	> 100
MET	> 100	> 100	> 100	35.59 (31.12 – 40.47)	> 100
TRA	> 100	69.69 (66.64 – 72.97)	> 100	58.66 (54.92 – 62.96)	> 100

In accordance with the European Directive EC 93/67/EEC (European Commission 1993), chemicals are classified on the basis of their EC_50_ values; those with an EC_50_ < 1 mg L^−1^ are considered “very toxic to aquatic organisms”, 1–10 mg L^−1^ are “toxic to aquatic organisms”, 10–100 mgL^-1^ are “harmful to aquatic organisms”, and > 100 mg L^−1^ are “not classified as harmful to aquatic organisms”. Based on this directive, most of the pharmaceuticals tested in our study for which a toxic effect was observed can be classified as harmful to aquatic environment. The highest toxic effect was observed for the antibacterial drug from the group of sulfonamides—SMZ. Since the EC_50_ values obtained in the tests with algae and duckweed were below 10 mg L^−1^, it can be classified as “toxic to aquatic organisms”. The high toxicity of SMZ to algae in comparison to luminescent bacteria and invertebrates was also described earlier (Ferrari et al. 2003; Isidori et al. 2005a; Minguez et al. 2016). On the other hand, the less toxic compounds were CRB, MET, and TRA. For CRB, toxic effect was only observed in the *L. minor* test (EC_50_ = 50.17 mg L^−1^), while for all other organisms the pharmaceutical was not toxic up to the concentration of 100 mg L^−1^, which is in agreement with the ecotoxicological data available in the literature (Cleuvers 2003; Ferrari et al. 2003; Kim et al. 2007; Donner et al. 2013; Minguez et al. 2016; Di Poi et al. 2018). Similarly, observed selective toxicity of MET towards green algae was reported before by other authors for *R. subcapitata* (Minguez et al. 2016), *Desmodesmus subspicatus* (Cleuvers 2003) and in our previous study for *Scenedesmus vacuolatus* (Maszkowska et al. 2014). Furthermore, we have proved that TRA can be recognized as harmful to *D. magna* (EC_50_ = 69.69 mg L^−1^) and *R. subcapiata* (EC_50_ = 58.66 mg L^−1^). The available ecotoxicological data for this compound is very limited. However, our observation is consistent with the data recently published by the group of Romanucci et al. (2019), who also did not observed any toxic effect of TRA towards *V. fischeri* at the concentration of 100 mg L^−1^ and documented the toxicity of TRA towards *D. magna* (EC_50, 24h_ = 88.5 mg L^−1^) and *R. subcapitata* (EC_50_ = 87.1 mg L^−1^). Very low toxicity of TRA was also observed in the other studies (Αntonopoulou and Konstantinou 2016; Αntonopoulou et al., 2020). Similarly as in our study, TRA was also not toxic to *V. fischeri* and *L. minor*; furthermore, the reported EC_50_ value in the *D. magna* immobilization test was the same (US Food and Drug Administration 1996). On the contrary, Le et al. (2011) evaluated the toxicity of TRA towards *D. magna* and determined EC_50_ at 170 mg L^−1^, which is more than two times higher than in our study.

Finally, based on the presented EC_50_ values, the investigated NSAIDs (such as IBU, DIC, and NAP) in our study can be classified as harmful to all tested aquatic organisms, while they did not pose any toxic effect to the soil bacteria *A. globiformis*. Although some ecotoxicological data for these compounds is already available, the reported EC_50_ values differ; hence, further studies are still needed. The EC_50_ value determined in our study in the *V. fischeri* test for DIC is in agreement with the results presented by Ferrari et al. (2003) and Czech et al. (2014). The observed toxicity of DIC towards *D. magna* is also consistent with literature data (Cleuvers 2003; Quinn et al. 2011; Minguez et al. 2016), in contrast to the determined toxicity towards *L. minor* (EC_50_ = 16.52 mg L^−1^)*,* which is almost three times higher than reported (EC_50_ = 47.6 mg L^−1^) by Quinn et al. (2011). The available data for IBU showed similar toxicity expressed in EC_50_ values in *L. minor* (Kaza et al. 2007) and *R. subcapitata* (Berrebaan et al. 2017) growth inhibition tests as well as in *V. fischeri* luminescence inhibition test (Halling-Sørensen et al. 1998) whereas the EC_50_ value in the test with *D. magna* was two times higher (Cleuvers 2003). The results of our study are also consistent with those reported in literature, low toxicity of NAP to *D. magna* (Cleuvers 2003; Minguez et al. 2016) and EC_50_ values obtained for duckweed (EC_50_ = 24 mg L^−1^ (Cleuvers 2003)) and algae (EC_50, 72h_ = 44.40 mg L^−1^ (Minguez et al. 2016) and EC_50, 96h_ = 31.82 mg L^−1^ (Isidori et al. 2005b)).

Furthermore, although differences in the sensitivity of the investigated organisms towards selected native forms of pharmaceuticals have been observed, it is not possible at this stage of knowledge to define the reasons. It might be only suspected that algae as well as higher plants are the most sensitive organisms in general, which may result from the specific mode of action of these pharmaceuticals. According to available literature, in case of NSAIDs, toxicity to non-target organisms can be explained by oxidative stress. Significant production of reactive oxygen and nitrogen species as well as increased lipid peroxidation was observed in chloroplasts isolated from *L. minor* at environmentally relevant concentrations (0.3–3.0 mg L^−1^) of DIC. Moreover, it was shown that higher concentrations of the pharmaceutical considerably affected photosynthetic processes that determine plant growth and development (Hájková et al. 2019). Another reason for the observed differences could be also the exposure time, as the test duration (72 h for algae and 7 days for duckweed) is longer compared to the other tests (30 min for *V. fischeri* and 48 h for *D. magna*). It must be also highlighted that investigated soil bacteria have not been affected by all of the investigated compounds in the concentrations up to 100 mg L^−1^.

Based on the obtained ecotoxicological data for the native forms of pharmaceuticals, the toxicity of transformation products was evaluated. All the results are presented in Tables 3, 4, 5, and 6.

**Table 3:** Comparison of *V. fischeri* luminescence inhibition after exposure to the native form (NF) and its TPs at the same concentration

Concentration [mg L^−1^]	NF	Luminescence inhibition [%]	TPs	Luminescence inhibition [%]
10	DIC	45	4-OH-DIC	38
10	IBU	42	2-OH-IBU CBX-IBU	4 2
25	NAP	51	*O*-DES-NAP	20
50	SMZ	46	N_4_-SMZ	8
100	CRB	42	CRB-Ep 10-OH-CBZ	30 N.A.
100	MET	3	MET-ACID	0
100	TRA	1	*O*-DES-TRA	0

*N.A.* not available

**Table 4:** Comparison of immobilization of *D. magna* after exposure to the native form (NF) and its TPs at the same concentration

Concentration [mg L^−1^]	NF	Immobilization [%]	TPs	Immobilization [%]
60	DIC	50	4-OH-DIC	8
50	IBU	52	2-OH-IBU CBX-IBU	0 8
70	NAP	32	*O*-DES-NAP	0
40	SMZ	48	N_4_-SMZ	0
100	CRB	24	CRB-Ep 10-OH-CRB	0 N.A.
100	MET	4	MET-ACID	0
70	TRA	44	*O*-DES-TRA	8

*N.A.* not available

**Table 5:** Comparison of growth inhibition of *L. minor* after exposure to the native form (NF) and its TPs at the same concentration

Concentration [mg L^−1^]	NF	Growth inhibition [%]	TPs	Growth inhibition [%]
15	DIC	33	4-OH-DIC	0
15	IBU	54	2-OH-IBU CBX-IBU	11 6
3	SMZ	49	N_4_-SMZ	17
50	CRB	49	CRB-Ep 10-OH-CRB	32 11
100	MET	5	MET-ACID	5
100	TRA	2	*O*-DES-TRA	0

**Table 6:** Comparison of growth inhibition of *R. subcapitata* after exposure to the native form (NF) and its TPs at the same concentration

Concentration [mg L^−1^]	NF	Growth inhibition [%]	TPs	Growth inhibition [%]
50	DIC	NA	4-OH-DIC	45
100	IBU	52	2-OH-IBU CBX-IBU	16 14
5	SULF	43	N_4_-SMZ	27
100	CRB	48	CRB-Ep 10-OH-CRB	37 15
100	MET	90	MET-ACID	0
100	TRA	93	*O*-DES-TRA	35

*N.A.* not available

The comparison of the determined luminescence inhibition (*V. fischeri* test) for all tested native forms and their TPs is presented in Table 3. Although it was observed that the toxicity of the tested TPs to *V. fischeri* was in general lower than its native form, it must be highlighted that TP of DIC was only slightly less toxic than its native compound (luminescence inhibition of 38% and 45%, respectively). It is also worth emphasizing that the toxicity of both compounds to luminescent bacteria was significant, as evidenced by a 40% inhibition of luminescence at a concentration of 10 mg L^−1^. Also, the biological activity of *O*-DES-NAP and 10-OH-CRB cannot be neglected. However, it might be concluded that only the selected TPs of metoprolol and tramadol do not pose any risk to *V. fischeri.* The lower toxicity of selected transformation products of TRA (including *O*-DES-TRA) towards these bacteria was also observed by other authors (Αntonopoulou and Konstantinou 2016; Αntonopoulou et al., 2020). The toxic effect of exposure to N_4_-SMZ and transformation products of IBU was also negligible. Majewsky et al. (2014) also reported similar trend—lower toxicity of N_4_-SMZ in comparison with its native form in the luminescence inhibition test; however, differences in the determined EC_50_ values were significant.

In the case of the *D. magna* immobilization test (Table 4), it was observed that all TPs were less toxic than their native forms. Taking into account the determined EC_50_ values for native forms and the observed effect caused by metabolites, *D. magna* turned out to be the least sensitive aquatic organism out of those selected for testing.

Furthermore, based on the comparison of the potency of the studied compounds to inhibit the growth of duckweed (Table 5), it was observed that MET-ACID and *O*-DES-TRA, similarly to their native forms, proved to be non-toxic. The highest tested concentration practically did not affect *L. minor* growth. However, both transformation products of IBU as well as 10-OH-CRB were much less toxic than IBU and CRB. Also, the toxicity of CRB-Ep was relatively high. The toxic effect caused by N_4_-SMZ is also worth emphasizing. Although the toxicity of the transformation product relative to the native form is more than three times lower, 17% inhibition of the growth of duckweed in concentrations as low as 3 mg L^−1^, proves its high potency to affect the organisms in the aquatic environment. This is also supported with the results for the algae test (Table 6), where the toxic effect of N_4_-SMZ to algae was also observed at similar concentration (5 mg L^−1^) of this compound. The obtained results indicate that both SMZ and its transformation products were the most toxic to algae and duckweed—the representative of higher aquatic plants. Both organisms as producers represent an important element of the trophic chain in the aquatic environment and inhibiting their growth due to chronic exposure to these compounds might have serious consequences for the entire ecosystem. Moreover, a relatively high effect towards green algae in comparison to native forms was observed at a concentration of 100 mg L^−1^ in the case of CRB-Ep (37%) and *O*-DES-TRA (35%).

Finally, in the test with soil bacteria *A. globiformis*, none of the studied drugs and their transformation products showed significant toxic effects—results obtained in the tested concentration range did not allow a determination of the EC_50_ value (Table 2). At the highest tested concentration, inhibition of bacterial growth was observed only for DIC (by 30%) and its transformation product—4-OH-DIC (by 14%). It should also be mentioned that the fact that these compounds cause a toxic effect in the acute toxicity test may suggest that chronic toxicity should be also evaluated to verify whether they may also pose a threat to soil microorganisms and influence the processes of biodegradation and circulation of elements.

Nevertheless, it must be highlighted that, although it seems that the threat posed by the investigated TPs is lower than for their native forms, it should not be neglected as in many cases their biological activity (although lower) was still observed and there is still a need to determine the real risk of their occurrence in the environment.

Furthermore, in our study, we have determined the toxicity of enantiomers of the two representative pharmaceuticals from the NSAIDs group—IBU and NAP (Table 7). The obtained EC_50_ values indicate two times higher toxicity of the R form of ibuprofen against algae and duckweed and almost three times higher toxicity of the R form of naproxen to luminescent bacteria. Different toxicological characteristics of enantiomers were suggested before (Nilos et al. 2011). Even though S-IBU and S-NAP are biologically active forms, the observed higher toxicity of R forms is in agreement with the results of significant number of enantiomer-specific toxicokinetic and toxicodynamic studies which have shown that these “inactive” enantiomers could be the stereoisomers that carry the toxic (side) effect (Nilos et al. 2011). However, in order to explain the possible reasons for the observed differences in the toxicity towards selected organisms, species-specific sensitivity (as a result of difference in biotransformation enzymes) should also be considered when dealing with chiral compounds (Nilos et al. 2011). Although, at this stage of our knowledge, it is not possible to indicate the exact reasons for the observed differences in toxicity of enantiomers, new ecotoxicological data presented in this study proves that chirality cannot be neglected in the ecotoxicological studies and determination of the risk posed by the pharmaceuticals and their TPs.

**Table 7:** EC_50_ values (mg L^−1^) (with confidence interval 2.5–97.5%) determined for the enantiomers selected for study of NSAIDs in the ecotoxicity tests

	EC_50_ [mg L^−1^]							
	*V. fischeri*		*D. magna*		*L. minor*		*R. subcapitata*	
	Isomer R	Isomer S	Isomer R	Isomer S	Isomer R	Isomer S	Isomer R	Isomer S
IBU	11.59 (10.43–12.66)	9.27 (8.98–9.56)	65.83 (59.54–72.54)	63.15 (59.66–67.66)	12.36 (10.80–14.07)	26.31 (22.25–31.66)	11.65 (10.21–13.26)	26.57 (22.50–31.88)
NAP	7.53 (7.11–7.99)	20.60 (19.50–21.75)	97.35 (91.90–104.95)	68.76 (54.64–90.98)	22.37 (20.76–24.04)	29.14 (27.19–31.40)	21.08 (19.64–22.59)	27.80 (25.67–30.05)

## Conclusions

New ecotoxicological data for seven native forms of pharmaceuticals and their 13 most important transformation products (including metabolites, degradation products and selected enantiomers) towards five different aquatic and soil organisms was provided. In general, it was observed that the toxicity of transformation products towards tested organisms was lower (if the toxic effect was observed in the investigated concentration range). However, in some cases, this toxicity differed slightly from this observed for the native form, like in the case of 4-OH-DIC towards *V. fischeri* (luminescence inhibition 38% for 4-OH-DIC and 45% for the native form) and CRB-Ep towards green algae (growth inhibition 49% for CRB and 37% for CRB-Ep) and duckweed (growth inhibition 49% for CRB and 32% for CRB-Ep). Moreover, two times higher toxicity of the R form of ibuprofen to algae and duckweed and almost three times higher toxicity of the R form of naproxen to luminescent bacteria was observed. This proves that the presence of metabolites and different degradation products of pharmaceuticals in the environment should not be neglected and further studies are still need in order to fully understand their environmental fate. However, it must be simultaneously highlighted that tested concentration ranges were of a few orders of magnitude higher than the concentrations of these compounds found in the environmental samples; therefore, in the further experiments rather chronic and mixture effects should be taken into the account. Nevertheless, the presented results are crucial in terms of the huge knowledge gap as well as global concern about the impact of the metabolites and degradation products of pharmaceuticals on the environment.

## Electronic supplementary material

ESM 1 (DOCX 481 kb)
