# Correction to: Ecotoxicity screening evaluation of selected pharmaceuticals and their transformation products towards various organisms

**DOI:** 10.1007/s11356-021-15410-3

**Published:** 2021-07-14

**Authors:** Łukasz Grabarczyk, Ewa Mulkiewicz, Stefan Stolte, Alan Puckowski, Magdalena Pazda, Piotr Stepnowski, Anna Białk-Bielińska

**Affiliations:** 1 Department of Environmental Analysis, Faculty of Chemistry, University of Gdańsk, ul. Wita Stwosza 63, 80-308 Gdańsk, Poland; 2 Institute of Water Chemistry, Technische Universität Dresden, 01062 Dresden, Germany

**Correction to: Environ Sci Pollut Res (2020) 27:26103–26114**

10.1007/s11356-020-08881-3

The correct Supplementary Material is presented in this paper.

## Supplementary Information

ESM 1 (PDF 458 kb)

