# Supplementary material for: Correction to: Ecotoxicity screening evaluation of selected pharmaceuticals and their transformation products towards various organisms
Source: Environ Sci Pollut Res Int. 2021 Jul 14;28(32):44582. doi: 10.1007/s11356-021-15410-3 (PMC8496651; doi:10.1007/s11356-021-15410-3)
Supplement: Supplementary file 1 — (PDF 458 kb) [file 11356_2021_15410_MOESM1_ESM.pdf]

Supplementary Material

For

Ecotoxicity screening evaluation of selected pharmaceuticals and their transformation products towards various organisms

Łukasz Grabarczyk[1], Ewa Mulkiewicz[1*], Stefan Stolte[2], Alan Puckowski[1], Magdalena Pazda[1], Piotr Stepnowski[1], Anna Białk-Bielińska[1]

[1]University of Gdańsk, Faculty of Chemistry, Department of Environmental Analysis, ul. Wita Stwosza 63, 80-308 Gdańsk, Poland

[2]Technische Universität Dresden, Institute of Water Chemistry, 01062 Dresden, Germany

*Corresponding author:

Ewa Mulkiewicz, ewa.mulkiewicz@ug.edu.pl, +48 58 523 51 90

Mathematical forms of each model associated with concentration-response curves (https://cran.r-project.org/web/packages/drfit/drfit.pdf)

The logistic model is widely used for sigmoidal dose response curves. For logistic model the four-parameter logistic function given by the formula (1) is used:

$$f\big(x,(b,c,d,e)\big) = c + \frac{d-c}{1+\exp\{b(\log(x)-\log(e))\}} \tag{1}$$

with four parameters *b, c, d, e*. The parameter *e* is also denoted ED_{50} and it is the dose producing the response half-way between the upper limit, *d*, and lower limit, *c*. The parameter *b* denotes the relative slope around *e*.

When the lower limit is fixed at 0 (c=0) and the upper limit is fixed at 1 (d=1) the two-parameter logistic function is obtained and the equation (1) has the form:

$$f\big(x,(b,e)\big) = \frac{1}{1+\exp\{b(\log(x)-\log(e))\}} \tag{2}$$

The equation (2) is used in logit fit.

For linear-logistic model Brain-Cousens's model is used given by the formula:

$$f(x,(b,c,d,e,f) = c + \frac{d+fx-c}{1+\exp\{b(\log(x)-\log(e))\}} \tag{3}$$

It is a five-parameter model, obtained by extending the four-parameter logistic model (1) adding parameter *f* to take into account inverse u-shaped hormesis effects.

Fixing the lower limit at 0 (c=0) yields the four-parameter model:

$$f(x,(b,d,e,f) = \frac{d+fx}{1+\exp\{b(\log(x)-\log(e))\}} \tag{4}$$

The equation (4) is used in linlogit fit.

The description of parameters obtained for the dose-response curves:

sigma - the square root of the estimated variance of the random error

a - for the linlogit model, this is the parameter e from formula (4); for the logit model, this is the ED50.

b - for the logit and linlogit models, these are the parameters b from equation (2) and (4)

c - only the linlogit fit produces a third parameter c, which is the parameter f from equation (4).

Table 1S. Composition of OECD TG 201 medium for *R. subcapitata*

Macroelements	Concentration [mg L^{-1}]	Microelements	Concentration [µg L^{-1}]
$NaHCO_3$	50.0	$FeCl_3 \times 6H_2O$	64.0
NH_4Cl	15.0	$Na_2EDTA \times 2H_2O$	100.0
$MgCl_2 \times 6H_2O$	12.0	H_3BO_3	185.0
$CaCl_2 \times 2H_2O$	18.0	$MnCl_2 \times 4H_2O$	415.0
$MgSO_4 \times 7H_2O$	15.0	$ZnCl_2$	3.0
KH_2PO_4	1.6	$CoCl_2 \times 6H_2O$	1.5
		$Na_2MoO_4 \times 2H_2O$	7.0
		$CuCl_2 \times 2H_2O$	0.01
**pH**	**8.1**		

Table 2S. Composition of pH-stabilised STEINBERG medium for *L. minor* prepared according to OECD 221

Macroelements	Concentration [mg L^{-1}]	Microelements	Concentration [µg L^{-1}]
KNO_3	350.0	H_3BO_3	120.0
$Ca(NO_3)_2 \times 4H_2O$	295.0	$ZnSO_4 \times 7H_2O$	180.0
KH_2PO_4	90.0	$Na_2MoO_4 \times 2H_2O$	44.0
K_2HPO_4	12.6	$MnCl_2 \times 4H_2O$	180.0
$MgSO_4 \times 7H_2O$	100.0	$FeCl_3 \times 6H_2O$	760.0
		$Na_2EDTA \times 2H_2O$	1500.0
**pH**	**5.5 +/- 0.2**		

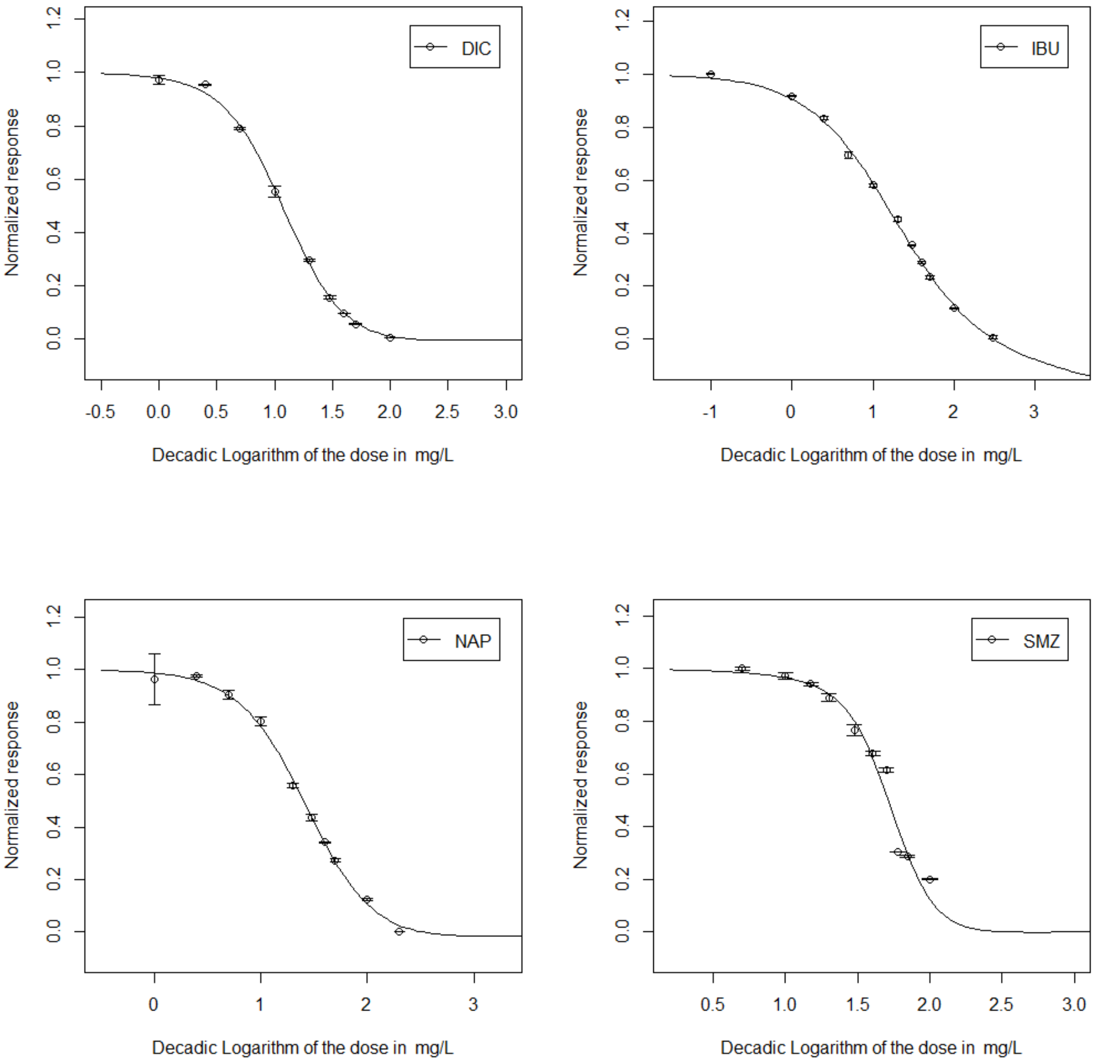

Fig. 1S. Dose-response curves obtained for studied native forms of pharmaceuticals in *V. fischeri* luminescence inhibition assay

Table 3S. Parameters describing the dose-response curves obtained in *V. fischeri* luminescence inhibition assay

**Compound**	**DIC**	**IBU**	**NAP**	**SMZ**
**Model type**	linlogit	linlogit	linlogit	linlogit
**logEC50**	1.065	1.175	1.401	1.714
**2.5%**	1.052	1.162	1.374	1.696
**97.5%**	1.079	1.188	1.427	1.732
**sigma**	0.017	0.014	0.038	0.051
**a**	1.065	1.175	1.401	1.714
**b**	1.666	0.822	1.374	2.894
**c**	-0.007	-0.003	-0.003	-0.003

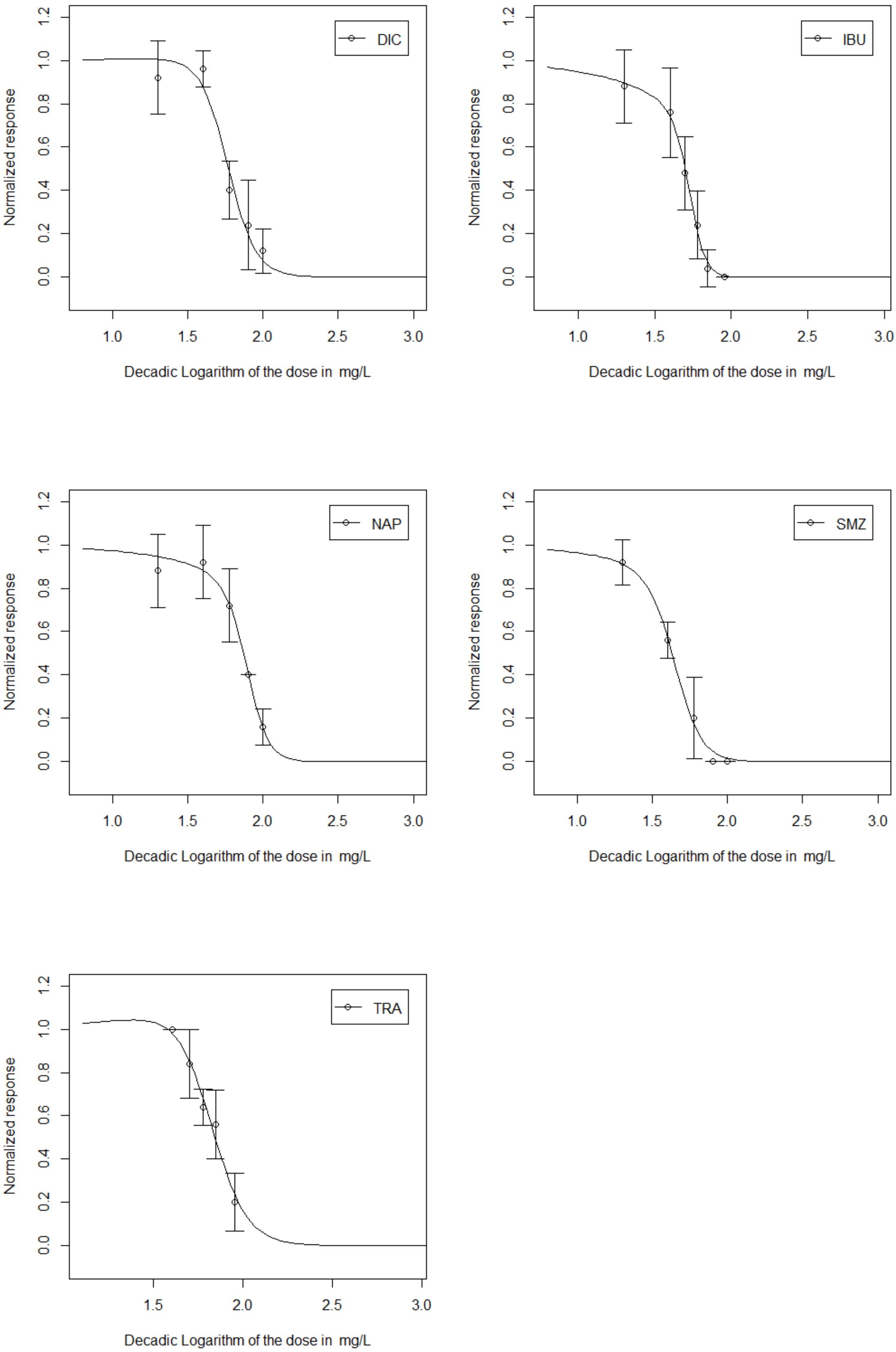

Fig. 2S. Dose-response curves obtained for studied native forms of pharmaceuticals in *D. magna* acute immobilization test

Table 4S. Parameters describing the dose-response curves obtained in *D. magna* acute immobilization test

**Compound**	**DIC**	**IBU**	**NAP**	**SMZ**	**TRA**
**Model type**	linlogit	linlogit	linlogit	linlogit	linlogit
**logEC50**	1.771	1.700	1.872	1.631	1.843
**2.5%**	1.742	1.677	1.845	1.604	1.824
**97.5%**	1.801	1.723	1.897	1.658	1.863
**sigma**	0.161	0.146	0.138	0.105	0.126
**a**	1.771	1.700	1.872	1.631	1.843
**b**	4.729	8.583	6.210	4.868	4.457
**c**	0.001	-0.005	-0.003	-0.004	0.002

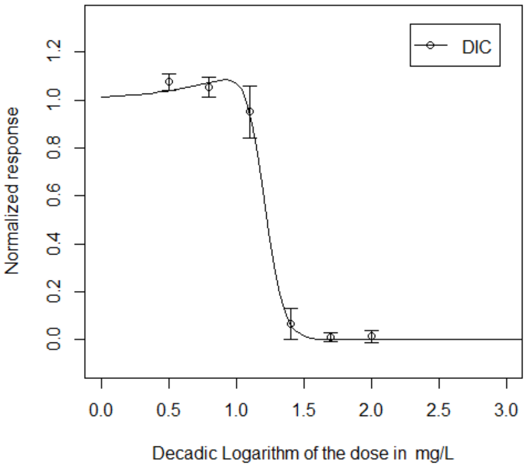

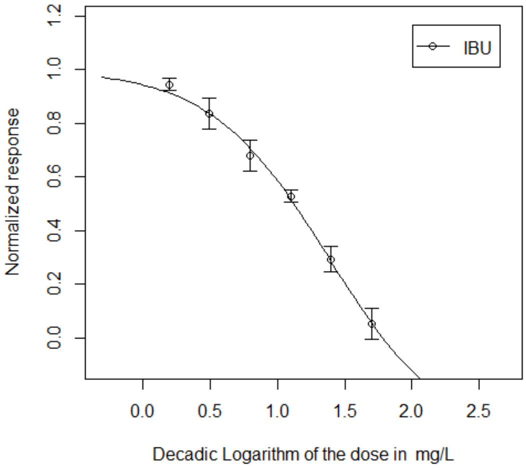

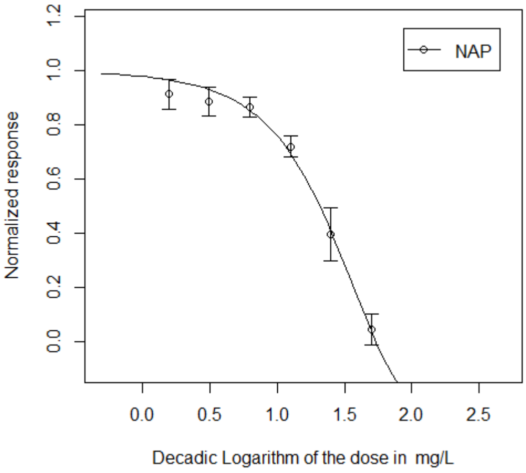

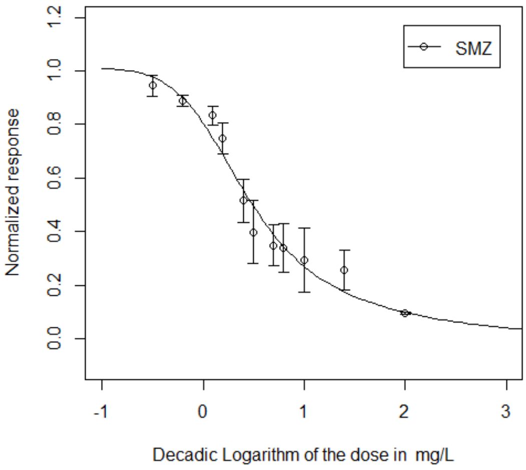

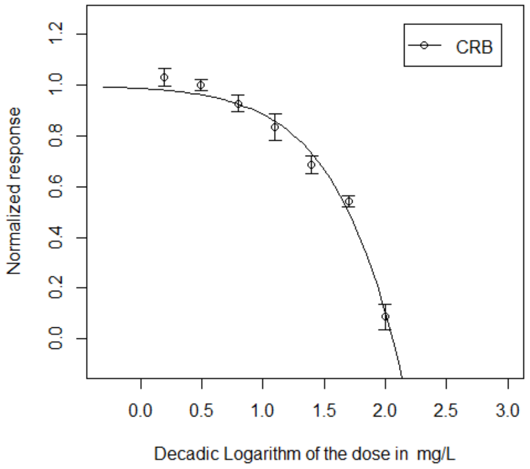

Fig. 3S. Dose-response curves obtained for studied native forms of pharmaceuticals in *L. minnor* growth inhibition test

Table 5S. Parameters describing the dose-response curves obtained in *L. minnor* growth inhibition test

**Compound**	**DIC**	**IBU**	**NAP**	**SMZ**	**CRB**
**Model type**	linlogit	linlogit	linlogit	linlogit	linlogit
**logEC50**	1.225	1.222	1.316	0.487	1.700
**2.5%**	1.199	1.084	1.276	0.419	1.667
**97.5%**	1.248	1.161	1.375	0.557	1.731
**sigma**	0.058	0.049	0.066	0.085	0.051
**a**	1.225	1.122	1.316	0.487	1.700
**b**	6.429	0.998	1.592	1.375	0.843
**c**	0.012	-0.016	-0.019	0.393	-0.009

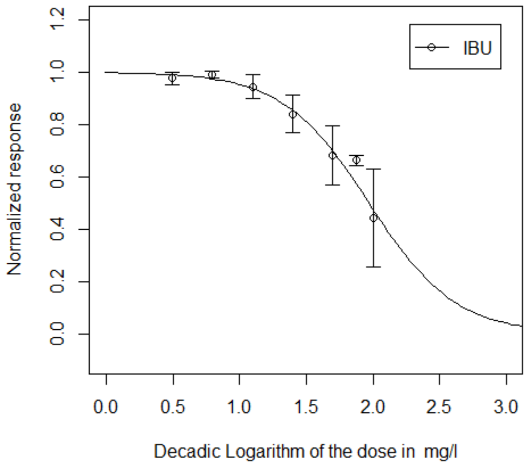

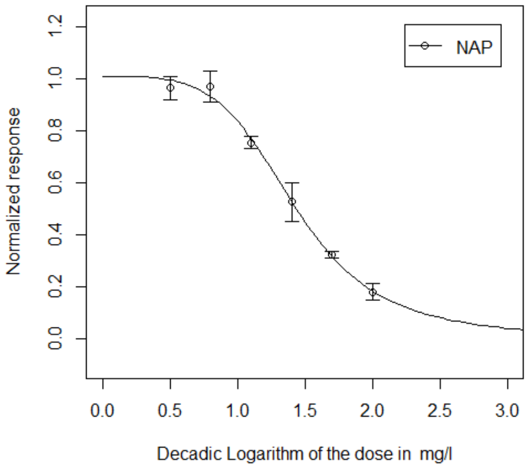

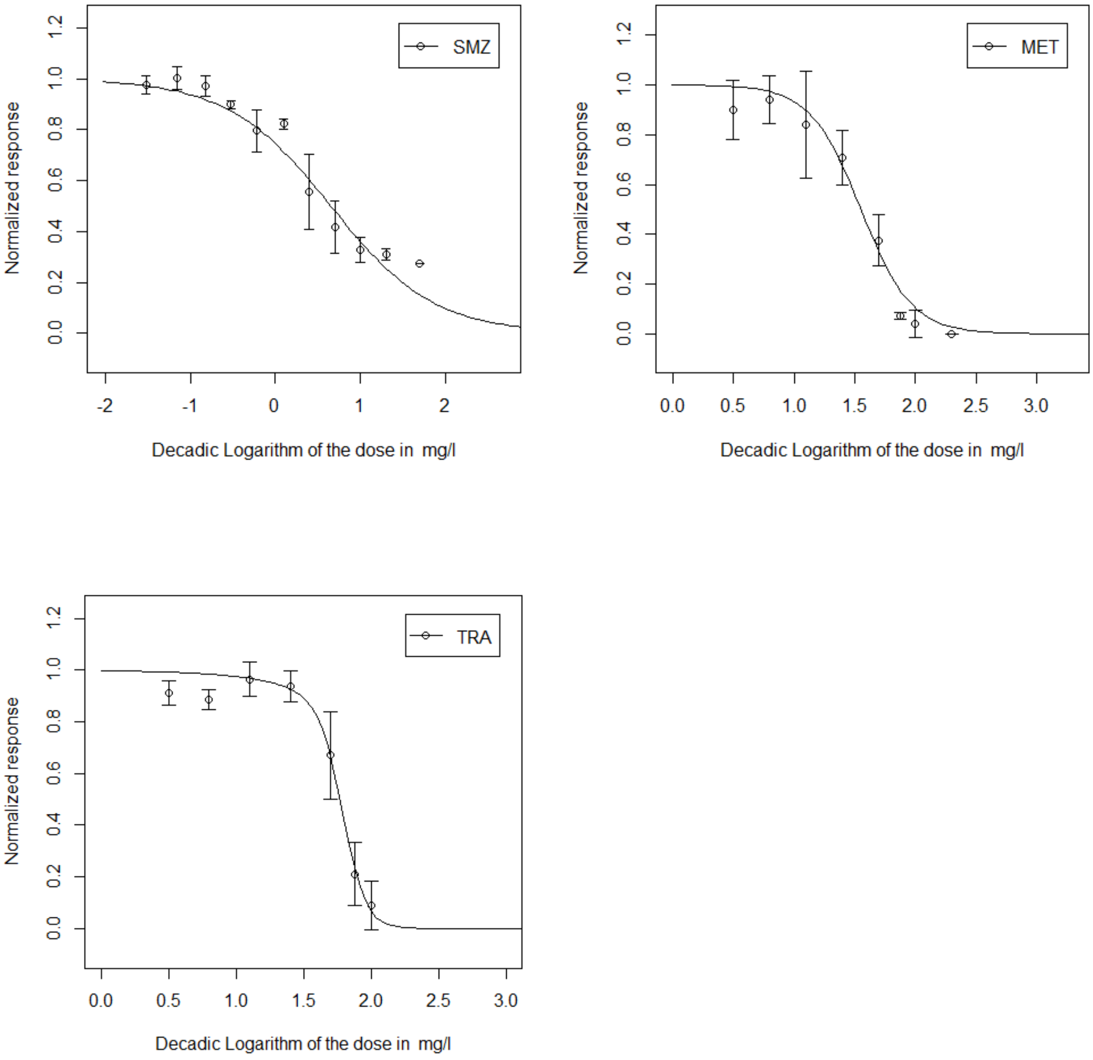

Fig. 4S. Dose-response curves obtained for studied native forms of pharmaceuticals in *R. subcapitata* reproduction inhibition test

Table 6S. Parameters describing the dose-response curves obtained in *R. subcapitata* reproduction inhibition test

**Compound**	**IBU**	**NAP**	**SMZ**	**MET**	**TRA**
**Model type**	logit	linlogit	logit	logit	linlogit
**logEC50**	1.970	1.433	0.639	1.551	1.768
**2.5%**	1.891	1.395	0.539	1.493	1.740
**97.5%**	2.087	1.473	0.742	1.607	1.799
**sigma**	0.101	0.049	0.088	0.129	0.106
**a**	1.970	1.433	0.639	1.551	1.768
**b**	0.323	1.561	0.585	0.209	5.015
**c**	-	0.024	-	-	-0.003

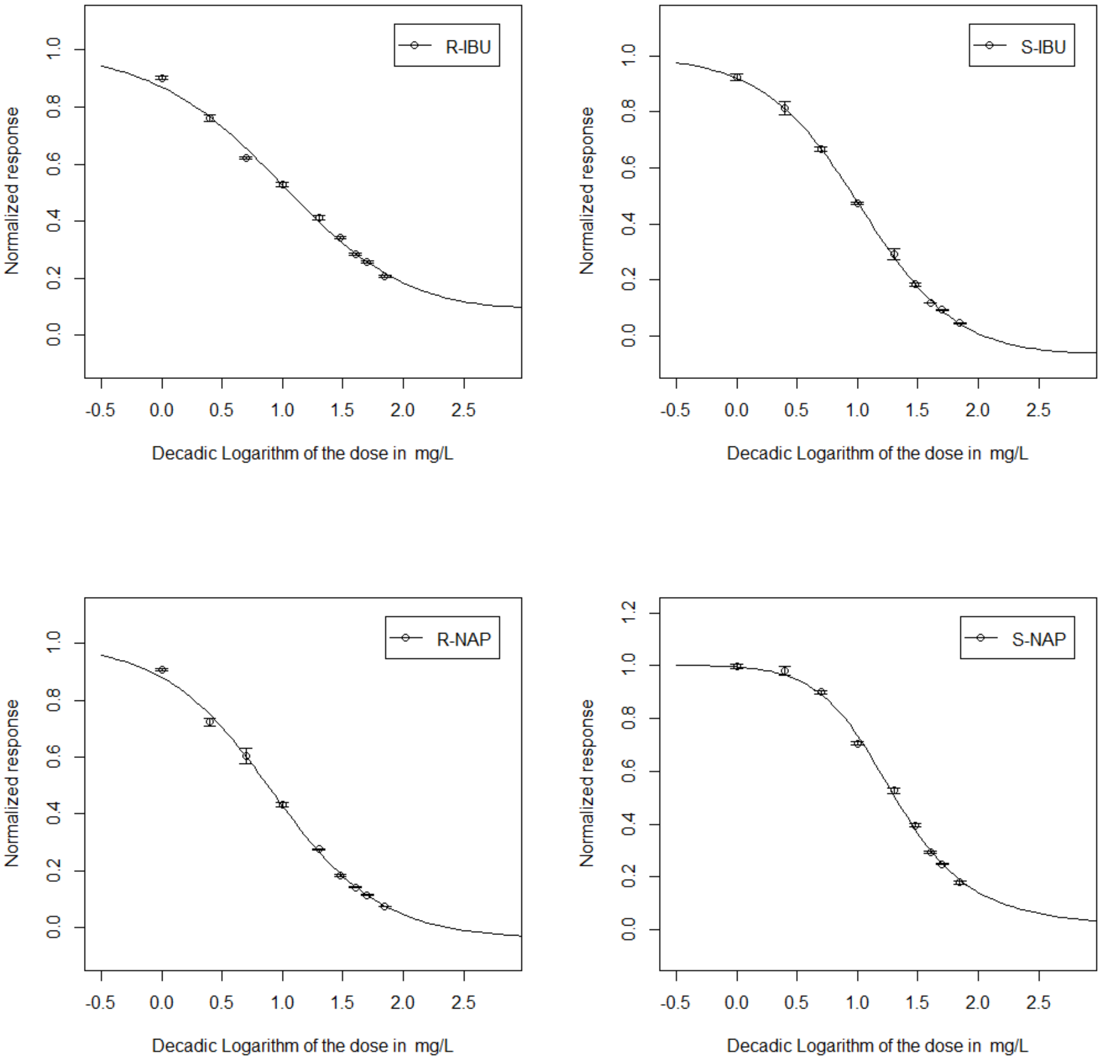

Fig. 5S. Dose-response curves obtained for studied native forms of pharmaceuticals in *V. fischeri* luminescence inhibition assay

Table 7S. Parameters describing the dose-response curves obtained in *V. fischeri* luminescence inhibition assay

**Compound**	**R-IBU**	**S-IBU**	**R-NAP**	**S-NAP**
**Model type**	linlogit	linlogit	linlogit	linlogit
**logEC50**	1.061	0.997	0.877	1.314
**2.5%**	1.018	0.953	0.852	1.290
**97.5%**	1.102	0.981	0.902	1.337
**sigma**	0.020	0.010	0.016	0.017
**a**	1.061	0.997	0.877	1.314
**b**	0.797	1.076	0.974	1.460
**c**	0.003	-0.009	-0.004	0.014

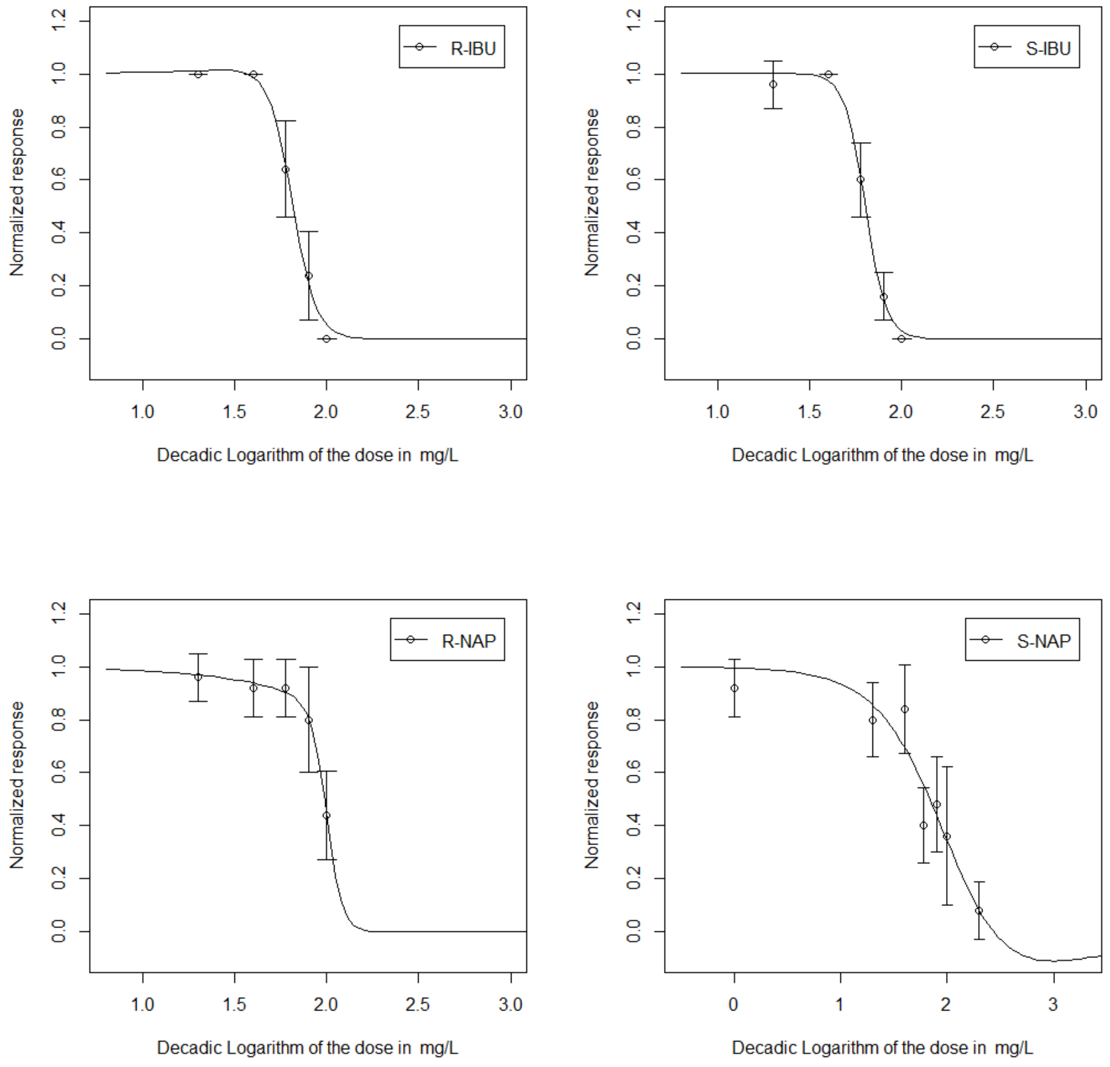

Fig. 6S. Dose-response curves obtained for studied native forms of pharmaceuticals in *D. magna* acute immobilization test

Table 8S. Parameters describing the dose-response curves obtained in *D. magna* acute immobilization test

Compound	R-IBU	S-IBU	R-NAP	S-NAP
**Model type**	linlogit	linlogit	linlogit	linlogit
**logEC50**	1.817	1.803	1.988	1.837
**2.5%**	1.775	1.773	1.963	1.738
**97.5%**	1.861	1.833	2.021	1.959
**sigma**	0.192	0.145	0.135	0.181
**a**	1.817	1.803	1.988	1.837
**b**	6.755	7.630	10.361	1.432
**c**	0.001	0	-0.002	-0.004

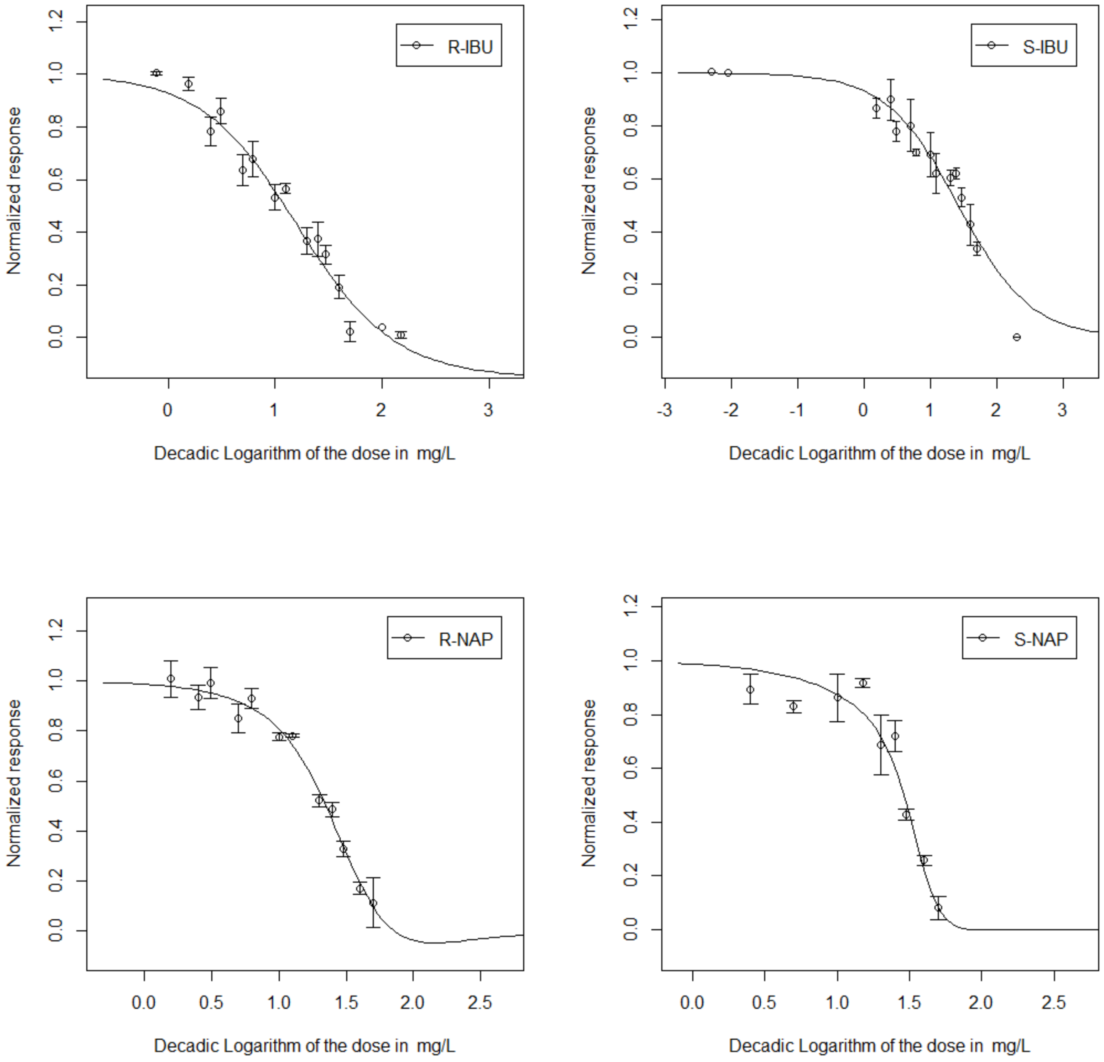

Fig. 7S. Dose-response curves obtained for studied native forms of pharmaceuticals in *L. minnor* growth inhibition test

Table 9S. Parameters describing the dose-response curves obtained in *L. minnor* growth inhibition test

**Compound**	**R-IBU**	**S-IBU**	**R-NAP**	**S-NAP**
**Model type**	linlogit	logit	linlogit	linlogit
**logEC50**	1.092	1.424	1.350	1.465
**2.5%**	1.033	1.352	1.317	1.434
**97.5%**	1.148	1.504	1.381	1.497
**sigma**	0.073	0.076	0.057	0.089
**a**	1.092	1.424	1.350	1.465
**b**	0.974	0.542	2.136	4.800
**c**	-0.009	-	-0.014	-0.012

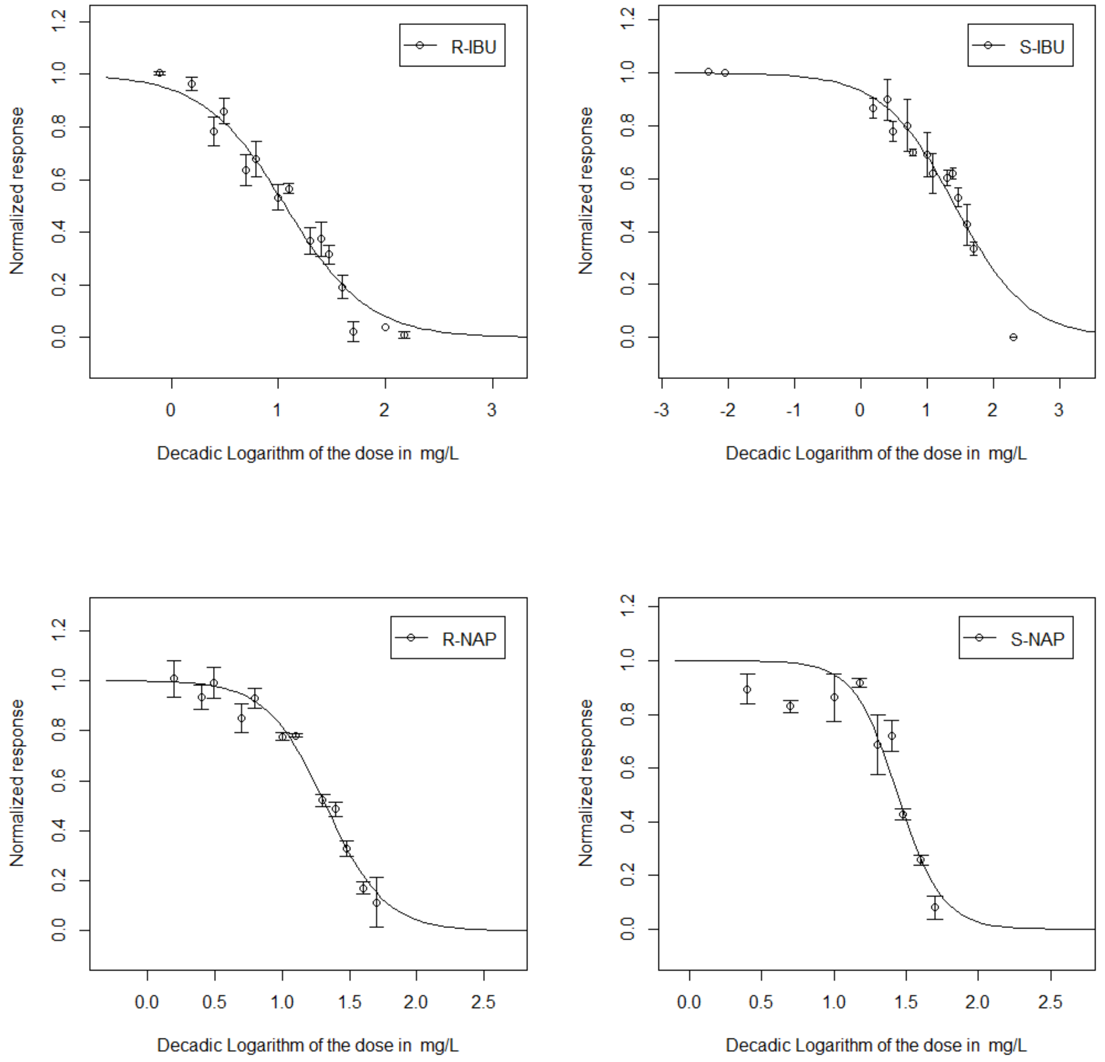

Fig. 8S. Dose-response curves obtained for studied native forms of pharmaceuticals in *R. subcapitata* reproduction inhibition test

Table 10S. Parameters describing the dose-response curves obtained in *R. subcapitata* reproduction inhibition test

**Compound**	**R-IBU**	**S-IBU**	**R-NAP**	**S-NAP**
**Model type**	logit	logit	logit	logit
**logEC50**	1.066	1.424	1.324	1.444
**2.5%**	1.009	1.352	1.293	1.410
**97.5%**	1.123	1.504	1.354	1.478
**sigma**	0.076	0.076	0.063	0.106
**a**	1.066	1.424	1.324	1.444
**b**	0.382	0.542	0.219	0.153
**c**	-	-	-	-
